# STRIKER: a spectral metadata repairing tool for expanding the comprehensiveness of spectral libraries

**DOI:** 10.1186/s13321-026-01150-4

**Published:** 2026-01-27

**Authors:** Ahmed Karam, Asmaa Ramzy, Taghreed Khaled Abdelmoneim, Maha Mokhtar, Nada A. Youssef, Aya Osama, Nabila Sabar, Sameh Magdeldin

**Affiliations:** 1 Proteomics and Metabolomics Research Program, Basic Research Department, Children’s Cancer Hospital Egypt 57357, Cairo, Egypt; 2 Physiology Department, Faculty of Veterinary Medicine, Suez Canal University, Ismailia, Egypt

**Keywords:** Untargeted metabolomics, Spectral library, Library repairing, Metadata, Python GUI, Adduct prediction, Adduct correction, Mass spectrometry

## Abstract

The expansion of untargeted metabolomics has made publicly accessible spectral libraries indispensable for metabolite annotation and machine learning applications. Enhancing the quality and consistency of these libraries is crucial for improving the accuracy of metabolite identification and training machine learning models. However, public spectral libraries often suffer from variability in user submissions, unintentional errors, and a lack of standardization. Existing metadata cleaning and normalization tools typically exclude spectra with incorrect or unsupported metadata rather than attempting to correct them, resulting in the loss of valuable spectral data and associated metabolites details. This study introduces STRIKER (SpecTRal lIbrary maKER), a repair tool specifically designed to address adduct metadata deficiencies using a distance-based metric and a deep learning model. STRIKER leverages advanced similarity-based approaches to predict adducts in spectra lacking adduct metadata. It corrects adduct-related errors and standardizes adduct formatting using a deep learning model based on the multi-layer perceptron (MLP) algorithm. STRIKER achieved 95–99% correct adduct matching and 98% adduct correction accuracy. These corrections substantially reduce the number of missing or unusable spectra and metabolites, thereby enhancing the accuracy of metabolite identification and improving data quality for machine learning applications. The tool also facilitates a convenient construction of the Human Metabolome Database (HMDB) spectral library by integrating data files from the HMDB website. Furthermore, it enables users to extract customized sub libraries from larger libraries, supporting tailored analyses for specific research objectives with percised search space. STRIKER is an open-source, user-friendly Python graphical interface designed to be accessible to researchers with minimal bioinformatics expertise. Available at the following repository under an MIT license: https://striker-gui.sourceforge.io.

**Scientific contribution**

The software is designed to preserve the maximum number of valid spectra in open mass spectral libraries, thereby supporting more comprehensive metabolite annotation in untargeted metabolomics. Its graphical user interface further facilitates the engagement of researchers without programming expertise, enabling them to enhance the quality and usability of spectral libraries.

**Supplementary Information:**

The online version contains supplementary material available at 10.1186/s13321-026-01150-4.

## Introduction

Spectral library matching for metabolite annotation is the dominant approach in untargeted metabolomics. In the absence of authentic standards, spectral library matching remains the gold standard for achieving confident metabolite annotation [1]. The spectral library, comprising an extensive repository of reference MS/MS spectra, provides the subject database for spectral matching against experimental MS/MS data [2]. HMDB, GNPS, MassBank, MoNA, and other open mass spectral libraries (OMSLs) constitute valuable community resources, offering freely available data that support both metabolite annotation and machine learning model development in metabolomics [3–6]. Unfortunately, most OMSLs exhibit inherent limitations, with some sources of weakness mitigated through existing pipelines while others remain unresolved and require further investigation. Variability in sample collection and preparation, instrumentation differences, analytical techniques used in the different experiments, parameters used in data acquisition, alongside the preprocessing workflows, heterogeneity in metadata nomenclature and the ununiformed reporting guidelines throughout the OMSLs, such as cases where an updated or more detailed representation of a compound alters the associated metadata (e.g., changes in the InChI identifiers), all these factors play a crucial role in enlarging the complexity and the inconsistency in data comparability during the integration across studies, hindering data sharing, reusing and reproducibility in metabolomics research [7–9].

A significant number of cleaning tools depend on external databases for metadata retrieval. Specifically, FragHub and matchms utilize RDKit to normalize chemical identifiers, including SMILES, InChI, and InChIKeys, primarily sourced from the ChEMBL database [10, 11]. For Instance, the recently developed Python-based graphical user interface, FragHub, enabled spectral library data integration and harmonization by resolving inconsistencies stemming from diverse analytical setups, library origins, specialized content, file formats, and metadata field standardization. FragHub managed to normalize chemical identifiers such as SMILES, InChI, InChIKey using internal dictionaries and RDKit. Several OMSLs formats (MSP, MGF, JSON, CSV, and XML) were converted into a unified, also classifying the spectra according to chromatographic techniques, mode of ionization and data origin whether predicted or experimental facilitated the data accessibility and integration reliability throughout the different OMSLs [12]. In addition to enhancing spectral libraries to broaden metabolite annotation capabilities, the acquisition of high-fidelity, error-minimized data is crucial for the development of robust machine learning models [13]. Matchms, an updated pipeline, provides crucial metadata cleaning capabilities, optimizing spectral library data for downstream analysis in tools like MZMine, MS-DIAL, and OpenMS, which are not intrinsically designed for spectral metadata cleaning [14–17].

Metadata inconsistencies can be rectified by leveraging complementary metadata, such as adduct correction based on precursor mass and SMILES representations. Salts appended to SMILES can also be corrected based on the exact mass. In certain cases, spectra are removed when discrepancies occur among the InChIKey, InChI, or SMILES representations referring to the same compound. Additionally, spectral entries exhibiting irrecoverable metadata deficiencies, including adduct and precursor m/z information, due to absence or format alteration, are subject to exclusion [12, 14, 18]. Precursor m/z serves as a pivotal identifier in metabolite annotation workflows employed by various software tools, including GNPS, MZmine3, MS-DIAL, and MetFrag [4, 15, 16, 19]. For instance, the Metabolome Annotation Workflow (MAW) initiates its analytical pipeline by retrieving spectral data from curated databases, including HMDB, MassBank, and GNPS, based on putative precursor mass values, thereby facilitating subsequent spectral matching and identification [20].

The noticeable absence of adduct form and precursor m/z metadata annotations is an issue in several spectral libraries. This deficiency, observed in a substantial quantity of spectra within downloadable Extensible Markup Language (XML) files from the HMDB website, can significantly impede data analysis and metabolite identification. Furthermore, it presents accessibility challenges by hindering the creation of spectral libraries containing reliable and readily manipulable spectra. In addition to missing adduct information, spectral libraries demonstrate variability in adduct form metadata, which impedes the reliable identification of adducts and subsequent precursor m/z determination. In fact, FragHub employed a comprehensive mapping dictionary of adducts and their calculated properties; however, pattern-matching approaches often yield suboptimal results [12]. For example, the adduct [M + H + H2O] + is not listed in the FragHub dictionary, whereas [M + H2O + H] + is included, even though both notations refer to the same chemical species. While recognizing the significant strides made by the scientific community in establishing open-source spectral libraries and mitigating access barriers, substantial data refinement remains essential to optimize these libraries for metabolomics data analysis and machine learning applications.

To address this gap, we present STRIKER, a tool designed to predict adducts for spectral libraries lacking adduct annotations. STRIKER supports multiple spectral similarity metrics, enabling users to select the most appropriate method for their data. In addition to adduct prediction, the presented tool corrects adduct formatting using a well-trained MLPClassifier. The tool is available as an open-source, user-friendly Python GUI.

## Implementation

The STRIKER workflow uses spectral matching techniques to implement an adduct annotation strategy for spectra lacking adduct information. Consequently, selecting an optimal matching algorithm necessitates rigorous evaluation across a diverse dataset of spectra from multiple spectral libraries. While STRIKER performs adduct form correction, its performance must be validated through comparative analysis with established state-of-the-art tools.

### Distance method benchmarking for adduct matching

#### Data collection

A dataset comprising the five most widely used public spectral libraries was collected to comprehensively benchmark prevalent distance methods for adduct matching (Table 1). These libraries were predominantly in MSP format, except for the HMDB library, which was originally available in XML format. However, the HMDB XML data lacked essential metadata fields such as InChIKey, SMILES, chemical formula, and exact mass. To address this deficiency, the missing information was retrieved from the HMDB website's Metabolite Browser in CSV format, merged with the XML-derived data, and subsequently converted to MSP format to ensure consistency across all libraries. Discrepancies between ionization modes and adduct annotations were manually corrected to fine-tune the experimental setup; however, these adjustments were applied exclusively to the HMDB library.

**Table 1:** Spectral libraries included in the benchmarking analysis

Spectral library name	Code	URL	File name	File format
GNPS	G	https://external.gnps2.org/gnpslibrary	ALL_GNPS_NO_PROPOGATED	MSP
HMDB	H	https://www.hmdb.ca/downloads	MS–MS Spectra Files (XML)—Experimental	XML
MassBank	MB	https://github.com/MassBank/MassBank-data/releases/tag/2024.11	MassBank_RIKEN-LC	MSP
MoNA	Mo	https://mona.fiehnlab.ucdavis.edu/downloads	LC–MS Spectra	MSP
MS-DIAL	MS	https://systemsomicslab.github.io/compms/msdial/main.html#MSP	ESI(+)-MS/MS from authentic standards ESI(−)-MS/MS from authentic standards	MSP

#### Assessment of metadata field reliability across libraries

To assess the reliability of metadata fields across public spectral libraries, we conducted systematic pairwise comparisons using random sampling. Four spectral libraries (G, MB, Mo, MS) were selected for evaluation. For each pairwise comparison, 5,000 spectra were randomly sampled from both the query and subject libraries. Using the CosineGreedy similarity method, all query spectra were matched against all subject spectra. This process was repeated 10 times to ensure robustness. Spectral pairs with a similarity score ≥ 0.95 were considered highly confident. From each iteration, the best match was selected for every query spectrum. To evaluate the reliability of individual metadata fields, we calculated the error rate—defined as the percentage of mismatches observed in other metadata fields when a specific field was identical between the query and subject spectra. For instance, we examined cases where the InChIKeys matched and then measured the mismatch rates across other fields such as name, SMILES, and InChI. This approach allowed us to quantify the consistency of each metadata field and assess their suitability for guiding method selection or spectrum comparison.

#### Preparing libraries for pairwise exclusion comparison

An intersectional set of InChIKeys common to all libraries (Experimental phase one) was required to establish a robust benchmark comparison. Given the inherent variability in spectra for identical InChIKeys, a pairwise exclusion comparative analysis was implemented, wherein each library was compared against the integration of the remaining libraries. The four spectral libraries, GNPS, MassBank, MoNA, and MS-DIAL, were initially integrated using FragHub with default parameter settings. Subsequently, individual libraries were extracted and recombined to generate integrated libraries (Figure S1, Additional file 1). The experimental phase two utilizes all InChIKeys from the HMDB library and performs a pairwise exclusion comparative analysis against the integrated set of the four spectral libraries.

#### Dynamic programming for spectra matching

We propose a new spectral matching method called Optimized Spectral Alignment (OSA) based on dynamic programming, designed to compare and align peaks. In addition to the gap penalty, a threshold-based similarity scoring scheme was incorporated to enhance alignment precision.

##### a. Preprocessing and input definition

The method takes as input two peak lists:

$$\begin{gathered} QS = \left( {\left( {qs\_mz_{1} ,qs\_{\mathrm{int}}_{1} } \right),\left( {qs\_mz_{2} ,qs\_{\mathrm{int}}_{2} } \right),...,\left( {qs\_mz_{m} ,qs\_{\mathrm{int}}_{m} } \right)} \right) \hfill \\ SS = \left( {\left( {ss\_mz_{1} ,ss\_{\mathrm{int}}_{1} } \right),\left( {ss\_mz_{2} ,ss\_{\mathrm{int}}_{2} } \right),...,\left( {ss\_mz_{n} ,ss\_{\mathrm{int}}_{n} } \right)} \right) \hfill \\ \end{gathered}$$

Given that query spectrum (*QS*) and subject spectrum (*SS*) may exhibit disparate lengths and contain peak values with varying degrees of proximity.

##### b. Dynamic programming framework

A dynamic programming (DP) matrix *dp* of size *(m+1) × (n+1)* is initialized to zero, with each element *dp[i,j]* representing the optimal alignment score between the first *i* elements of *QS* and the first *j* elements of *SS*.

The recurrence relation governing the score computation is:

$$dp[i,j] = \max \left\{ {\begin{array}{*{20}l} {dp[i - 1,j - 1] + final\_score} \hfill & {(match \, or \, mismatch)} \hfill \\ {dp[i - 1,j] + gap\_penalty} \hfill & {(gap \, in \, subject)} \hfill \\ {dp[i,j - 1] + gap\_penalty} \hfill & {(gap \, in \, query)} \hfill \\ \end{array} } \right.$$

where the* final_score* introduces a novel threshold-based scoring scheme:

$$final\_score = \left\{ {\begin{array}{*{20}l} {{\mathrm{int}} ensity\_score,} \hfill & {if \, |qs\_mz_{i} - ss\_mz_{j} | \le mz\_tolerance} \hfill \\ { - 1,} \hfill & {otherwise} \hfill \\ \end{array} } \right.$$

with a predefined threshold *mz_tolerance* = *0.005*, which accounts for minor variations in m/z values. The *gap_penalty* is set to −0.1, which is intentionally smaller in magnitude than the mismatch penalty, reflecting the lower likelihood and importance of gap events in the alignment process.

The use of intensities is optional; when incorporated, the *intensity_score* is computed based on the following criteria:

$${\mathrm{int}} ensity\_score = \frac{1}{{1 + \left( {local\_cityblock} \right)^{2} }}$$

Therefore, the highest score is achieved when the differences between intensities are minimized. These differences are estimated using the absolute difference method, as defined below:

$${\mathrm{local}}\_{\mathrm{cityblock}} = \left| {{\mathrm{qs}}\_{\mathrm{int}}_{i} - {\mathrm{ss}}\_{\mathrm{int}}_{j} } \right|$$

If intensities are not used, the intensity score is uniformly set to one, and the overall score is determined solely by whether the m/z values fall within the specified tolerance threshold.

In cases where multiple matching results yield the same dynamic programming score, we employ the city-block distance as a secondary criterion to determine the optimal match.

$${\mathrm{global}}\_{\mathrm{cityblock}} + = \left| {{\mathrm{qs}}\_{\mathrm{mz}}_{i} - {\mathrm{ss}}\_{\mathrm{mz}}_{j} } \right|$$

The key distinction is that the differences between the m/z values will be calculated using the city-block distance method across the entire DP matrix, without the application of a specified tolerance.

#### Comparative evaluation of distance metrics for adduct prediction using pairwise exclusion comparison

A comparative performance analysis of spectral matching methods was conducted using the prepared libraries, utilizing ten distance methods from the SciPy package, the high-performance tools matchms and msentropy, and our developed spectral matching algorithm, OSA (Table 2) [21]. The comparison methodology primarily involves retrieving spectra from the subject library that match the query InChIKey. Subsequently, spectral matching is performed between spectra exhibiting congruent ion modes, and the optimal match is determined by selecting either the highest similarity score or the lowest distance score, depending on the respective distance or similarity metric applied. A match is defined by either concordance in adduct forms or a minimal deviation in precursor m/z (≤ 0.5 Da) between the query and subject spectra. In cases where neither criterion is met—i.e., there is no alignment in adduct forms nor acceptable precursor m/z deviation—the comparison is classified as a mismatch. The match rate is calculated as the percentage of matched spectra relative to the total number of query spectra. The comparison was performed at two levels: the first level considered only the m/z values, while the second level incorporated both m/z values and intensities.

**Table 2:** List of packages used in the benchmark

Method name	Package name
Entropy Similarity	Msentropy
CosineGreedy	Matchms
Optimal Spectra Alignment (OSA)	STRIKER
City Block	SciPy
Braycurtis	SciPy
Canberra	SciPy
Euclidean	SciPy
Sqeuclidean	SciPy
Minkowski	SciPy
Chebyshev	SciPy
Correlation	SciPy
Cosine	SciPy
Jensenshannon	SciPy

### MLP-Based correction of adduct forms

A Multi-Layer Perceptron (MLP) classifier was employed to perform adduct form correction in spectral data by training on labeled datasets. These datasets were derived from adduct masses extracted from the online repository hosted by the Fiehn Lab, the comprehensive mapping dictionary integrated within the FragHub source code, and all available adduct form annotations within OMSLs [22]. Subsequently, positional permutations of ions within each adduct were performed, and labels for both data provenance sources were manually generated. Input adduct forms underwent a preprocessing toward normalizing adduct structures, handling brackets, ions, and delimiters. The processed text was then converted into numerical features using TF-IDF (term frequency–inverse document frequency) vectorization (character-level, n-gram range 1–3). A single-layer MLP classifier (200 hidden units, max 500 iterations) was trained on this representation and evaluated using accuracy and classification reports.

**Fig. 1:**
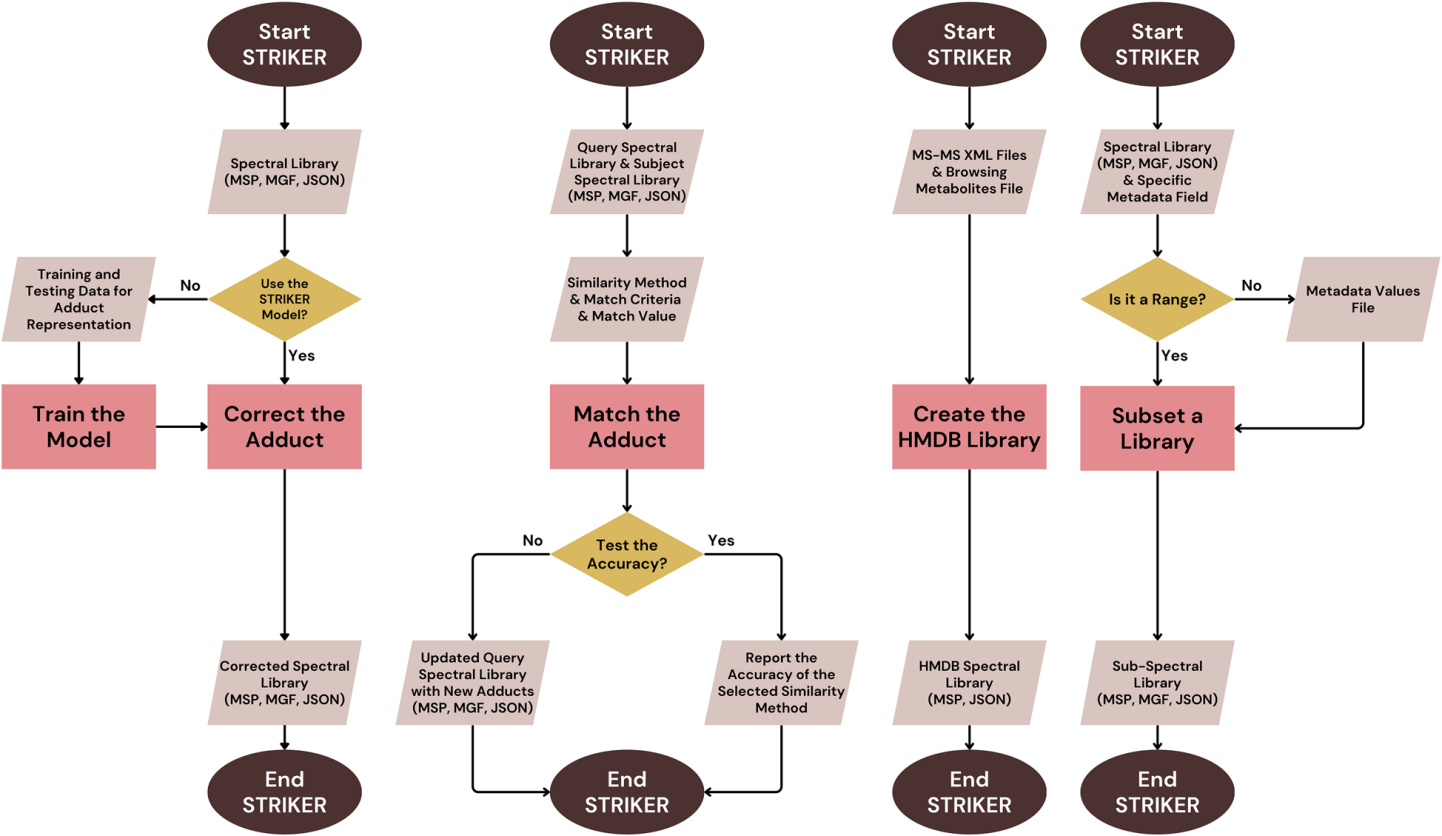
A schematic flowchart illustrating the tasks executed by STRIKER. STRIKER offers four core services, illustrated from left to right in the figure: Correct the Adduct, Match the Adduct, Create the HMDB Library, and Subset a Library. In the Correct the Adduct workflow, STRIKER employs an MLPClassifier deep learning model to detect, correct, and standardize errors in adduct annotations. Users can choose to apply the STRIKER model developed and validated in this study or train and evaluate a new model using their own data. The Match the Adduct workflow enables adduct prediction by comparing spectra from a query library to a large subject library using various similarity metrics. STRIKER evaluates each method’s performance by comparing spectra with known adducts in the query library to their matches in the subject library. This benchmarking step enables the user to identify the most accurate similarity method, which is subsequently applied to predict adducts in spectra with unknown adduct annotations. In the Create the HMDB Library workflow, STRIKER parses and integrates metadata from HMDB XML files along with compound information from the metabolite CSV file to construct a standardized HMDB spectral library. Finally, the Subset a Library workflow allows users to extract a subset of spectra from a large spectral library based on specified metadata values. For continuous fields such as precursor m/z or exact mass, users can specify ranges; for categorical metadata, exact matches are used for subsetting

STRIKER was developed in Python, utilizing matchms parsers for reading and writing spectral files, and PySide6 for constructing the GUI. A schematic flowchart on STRIKER is illustrated in Fig. 1.

## Results

STRIKER, a Python-based GUI tool, was developed to address limitations in OMSLs that impede metabolite annotation in untargeted LC–MS metabolomics studies. In this study, five OMSLs were utilized to quantitatively assess the prevalence of missing adduct annotations and heterogeneity across the OMSLs.

We began by evaluating the reliability and consistency of commonly used compound identifiers across public spectral libraries. Notably, we observed a 7.4% mismatch rate in SMILES strings despite identical InChIKeys, highlighting a key limitation of non-canonical SMILES representations (Figure S2.A, Additional file 1). A similar trend was seen when using InChI as the matching criterion, reinforcing the well-established equivalence between InChI and InChIKey in representing chemical identity. These findings emphasize that while InChIKey and InChI are robust identifiers, SMILES can lead to apparent inconsistencies if not properly canonicalized. Conversely, when SMILES strings were identical, the mismatch rate for InChI and InChIKey dropped to nearly zero, underscoring the greater consistency of InChI-based identifiers. Taken together, these observations support the prioritization of InChI and InChIKey over SMILES in metadata curation and compound alignment workflows, particularly for adduct prediction tasks. Importantly, the analysis also revealed that using molecular formulas or compound names as identifiers results in substantially higher mismatch rates across other metadata fields. This outcome is expected, given that different compounds can share the same molecular formula, and compound names are prone to ambiguity due to inconsistent naming conventions and synonyms. Although the use of SMILES and molecular formulas may yield less accurate results compared to the InChIKey in the adduct prediction task, the STRIKER GUI provides flexibility by supporting InChIKey, SMILES, or molecular formula as input identifiers.

It is not feasible to rely on a fixed similarity score threshold as a reliable indicator of correct compound matches. As shown in Figure S2.B, Additional file 1, a substantial mismatch rate was observed even among spectral pairs with similarity scores between 0.95 and 1.0. This finding highlights that highly similar spectra can, in fact, correspond to different compounds. The analysis was conducted without applying any filters based on ion mode or InChIKey; spectra were randomly selected and compared solely on the basis of their similarity scores. These results underscore the limitation of using high similarity scores alone for match validation and emphasize the need to incorporate metadata filtering and structural information for more accurate spectral matching.

**Fig. 2:**
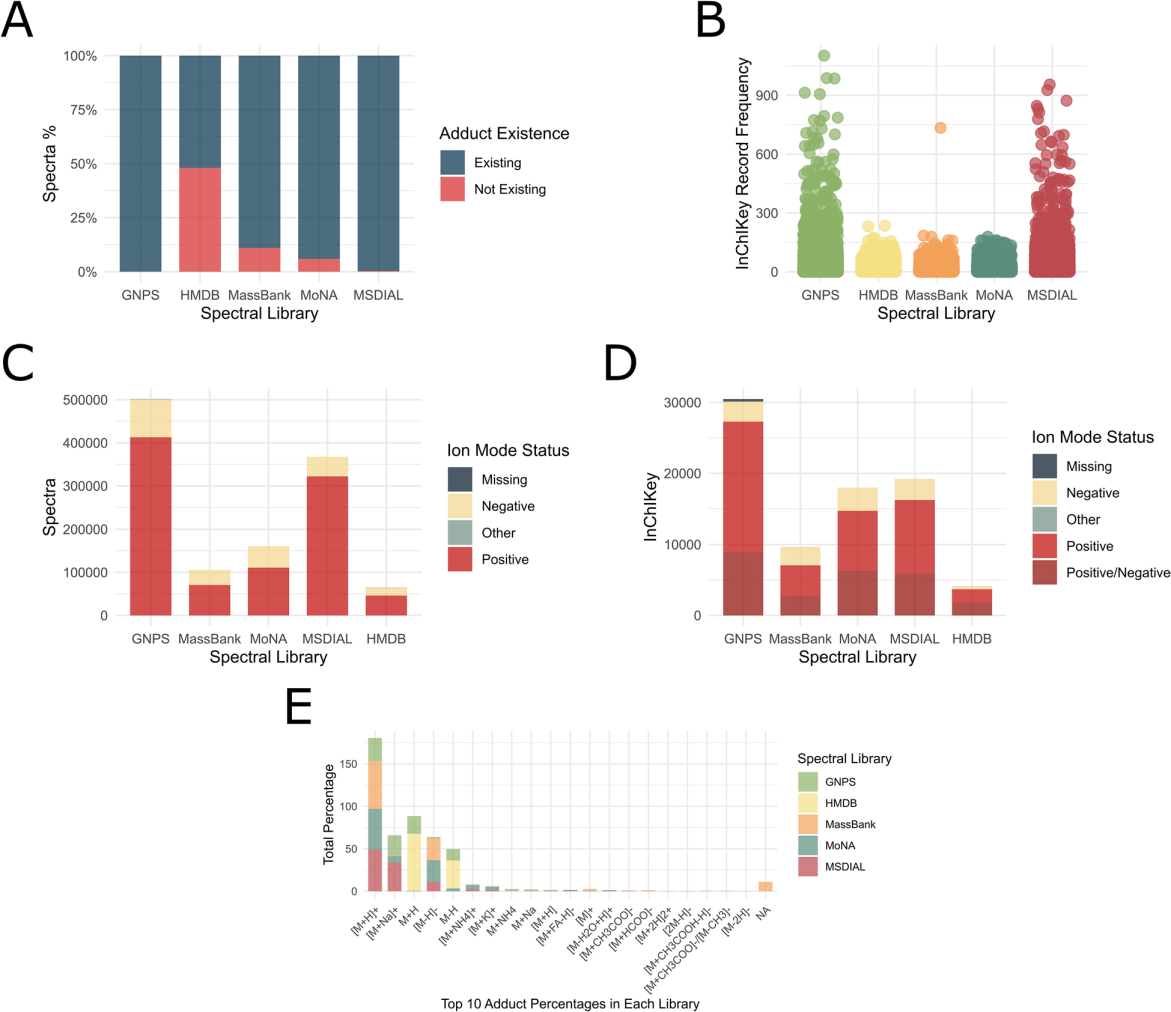
A comprehensive statistical overview of the OMSLs: **A** Stacked bar plot showing the proportion of spectra with existing versus missing adduct type metadata across five spectral libraries. The plot highlights the completeness of adduct annotations in GNPS, HMDB, MassBank, MoNA, and MSDIAL. **B** Beeswarm plot showing the distribution of spectrum counts per unique InChIKey across five spectral libraries. Each point represents a distinct InChIKey, positioned along the y-axis by the number of associated spectra and along the x-axis by the spectral library of origin. This visualization highlights variation in the representation and redundancy of InChIKeys among GNPS, HMDB, MassBank, MoNA, and MSDIAL. **C** Stacked bar plot illustrating the distribution of ionization modes across five spectral libraries. Each bar represents the total number of spectra per library, partitioned by ionization mode (positive, negative, missing, and other). **D** Stacked bar plot showing the distribution of ionization mode metadata at the InChIKey level across five spectral libraries. Each bar represents the total number of unique InChIKeys, categorized by ionization mode assignment: Positive only, Negative only, Positive/Negative (observed in both modes), other, and Missing. Unlike figure C, which reflects spectrum-level ion mode counts, this figure highlights how individual chemical identifiers are distributed across ionization modes, revealing the extent to which compounds are captured under single or multiple ionization conditions in each library. **E** Stacked bar plot showing the distribution of the most frequently observed adduct types across spectral libraries. Each bar corresponds to a specific adduct type and is segmented to show the proportional representation of that adduct across the individual spectral libraries. This figure highlights both shared and library-specific adduct forms

The incidence of missing adducts in GNPS and MS-DIAL spectra was determined to be less than 0.05% (approximately 250 spectra) and 0.4% (approximately 1400 spectra), respectively, representing a minor proportion of their spectral content (Fig. 2.A). Conversely, MoNA and MassBank exhibited higher proportions of missing adducts, reaching 5.9% and 11%, respectively. Interestingly, HMDB library demonstrated the most significant deficiency in adduct annotations within its experimental spectra, reaching 48%, encompassing over 31,200 spectra.

The average spectra frequency per InChIKey was similar across libraries, with MS-DIAL showing the highest (19.1), followed by HMDB (16.4), GNPS (14.7), MassBank (10.9), and MoNA (8.8). Despite these comparable averages, both GNPS and MS-DIAL include InChIKeys with more than 900 associated spectra. However, such high-frequency cases are confined to the final third of the dataset and represent a minor fraction relative to the total number of InChIKeys in each library (Fig. 2.B).

The ion mode constitutes a critical metadata that is imperative for inclusion in any comparative spectral annotation. Given the fact that peak lists for identical InChIKeys exhibit significant variation when all metadata except ion mode are conserved, its incorporation as a matching criterion is essential for accurate spectral comparison. Our analysis reveals that GNPS has 1,661 spectra (0.33%) lacking ion mode metadata, and 353 unique InChIKeys (1.17%) (Fig. 2.C&D). Similarly, the HMDB exhibits a deficit of ion mode metadata in 199 spectra (0.30%), affecting 14 unique InChIKeys (0.34%). These findings demonstrate a low overall prevalence of missing ion mode metadata, thereby supporting its reliability as a key parameter in spectral matching algorithms. As for the adduct form, focusing on the prevalent [M + H]^+^ and [M-H]^−^ ions, our investigation revealed variations in their representation across different OMSLs. Specifically, MS-DIAL, MoNA, and HMDB predominantly maintain a singular annotated form for each of these adducts, with a lack of inter-library standardization of adduct form (Fig. 2.E). In contrast, GNPS and MassBank contain multiple annotated forms for the same adduct, alongside the presence of less frequent but diverse adduct forms. Therefore, it is crucial to implement a normalization strategy for this metadata to ensure uniformity across disparate libraries and to prevent information loss during normalization procedures utilizing currently available tools.

**Fig. 3:**
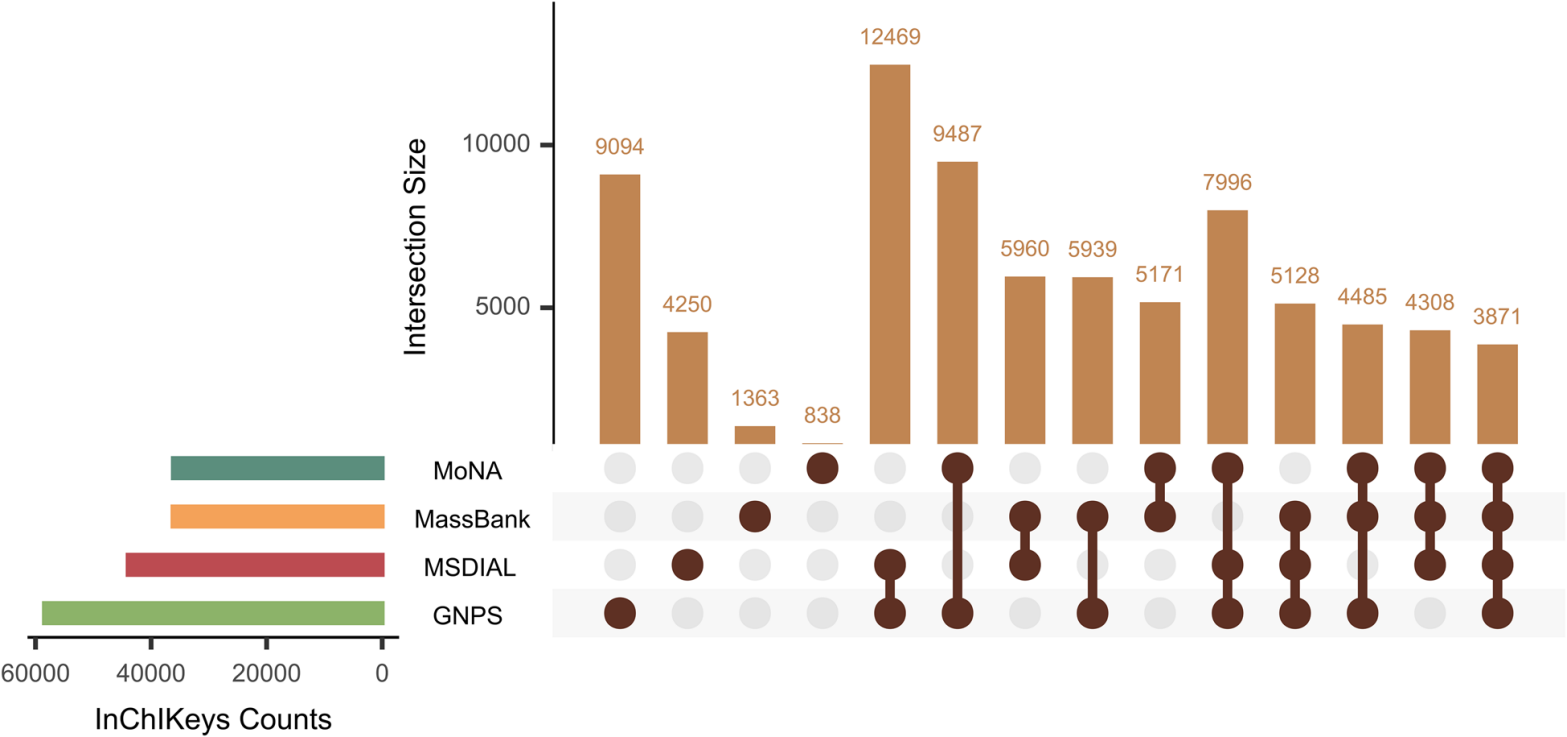
UpSet plot illustrates the intersecting sets among the four OMSLs. The number of shared InChIKeys across all OMSLs is 3871

Accurate prediction of adduct metadata is crucial for maintaining the comprehensiveness of spectral libraries, preventing the inadvertent exclusion of valuable spectra and associated metabolites. To recognizing this issue, a rigorous experimental design was implemented, leveraging multiple spectral libraries with harmonized metadata and common compounds for comparative analysis. The experimental design also incorporated a benchmark evaluation of prevalent spectral matching methodologies. FragHub was utilized to integrate the GNPS, MassBank, MoNA, and MS-DIAL spectral libraries to ensure seamless data exchange and integration. Subsequently, these libraries were partitioned into separate libraries and integrated libraries, as previously described, to evaluate the efficacy of the matching methods rigorously. The intersection of InChIKeys shared across all four libraries was selected as the basis for this initial experiment, which aimed to determine the percentage of correct matches for adduct or precursor m/z when querying a defined subset of InChIKeys within one library against the remaining three (Fig. 3). While the number of unique InChIKeys was consistent across the experimental setup, the inherent variability in the number of associated spectra within each library presented a significant analytical challenge for the robust evaluation of the matching methods. In addition to the aforementioned, an experiment was implemented to conduct a comprehensive spectral comparison by matching all InChIKeys within the HMDB library that contained adduct metadata against the unified library resulting from the integration of the four libraries (FragHub library).

**Fig. 4:**
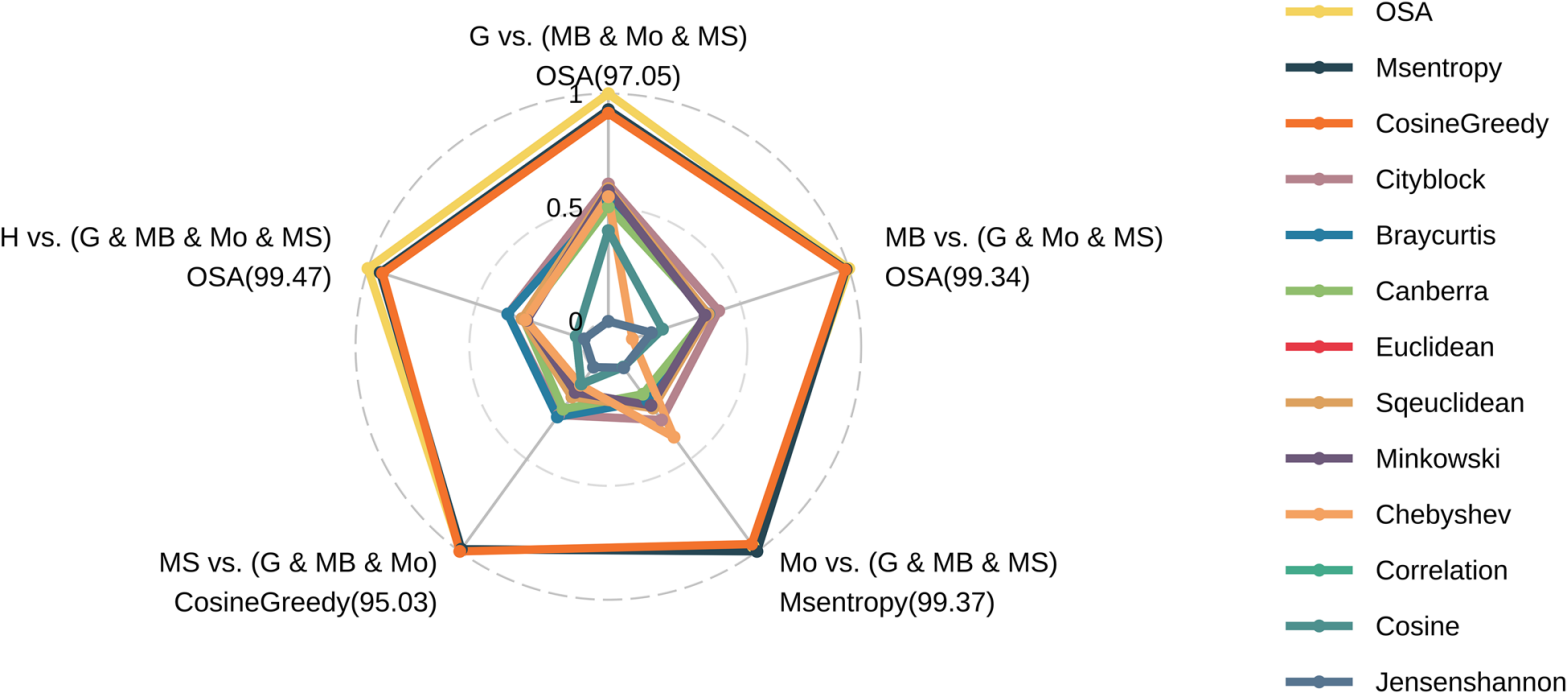
Radar plot illustrates the accuracy of each distance metric across various comparisons at the first level (m/z value). The scale ranges from 0 to 1, representing the minimum to maximum accuracy values within each comparison, thereby enhancing the visualization of subtle differences due to the overall similarity of the results

The results from the first level of comparison, which the usage of m/z values, demonstrated the superior performance of the OSA algorithm, exhibiting an average correct match rate of 98.04% (Additional file 2) and achieving the highest rate in three out of five comparisons (Fig. 4). Notably, OSA exhibited exceptional performance in the HMDB library versus the FragHub library comparison, yielding the highest correct match rate of 99.47%. Entropy Similarity secured the second place with an average correct match rate of 97.98%, demonstrating a marginal superiority in one out of five comparisons. CosineGreedy followed closely, presenting a minimal difference compared to Entropy Similarity, with an average correct match rate of 97.97%.

**Fig. 5:**
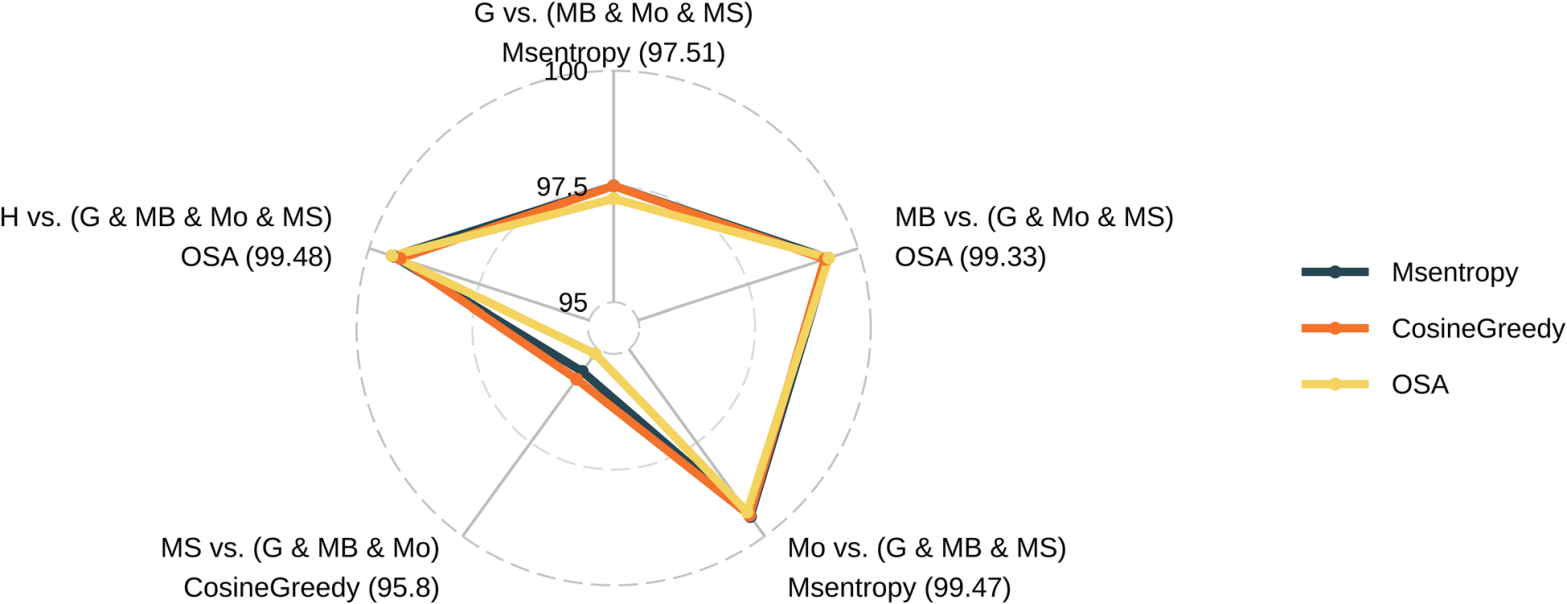
Radar plot depicting the accuracy of each distance metric across various comparisons at the second level (m/z and intensity values). The percentages were not normalized, as the lowest observed match rate exceeded 95%

For the second level of comparison, which incorporated both m/z values and intensities, Entropy Similarity, CosineGreedy, and OSA were employed, as these methods are specifically designed to utilize intensity information. At this level, Entropy Similarity achieved the highest average match rate at 98.27% (Additional file 3), followed closely by CosineGreedy at 98.26%, and OSA at 98.10% (Fig. 5). Notably, OSA outperformed all other methods in both the first and second level comparisons, achieving a match rate of 99.48% in the comparison between the HMDB and FragHub library.

**Fig. 6:**
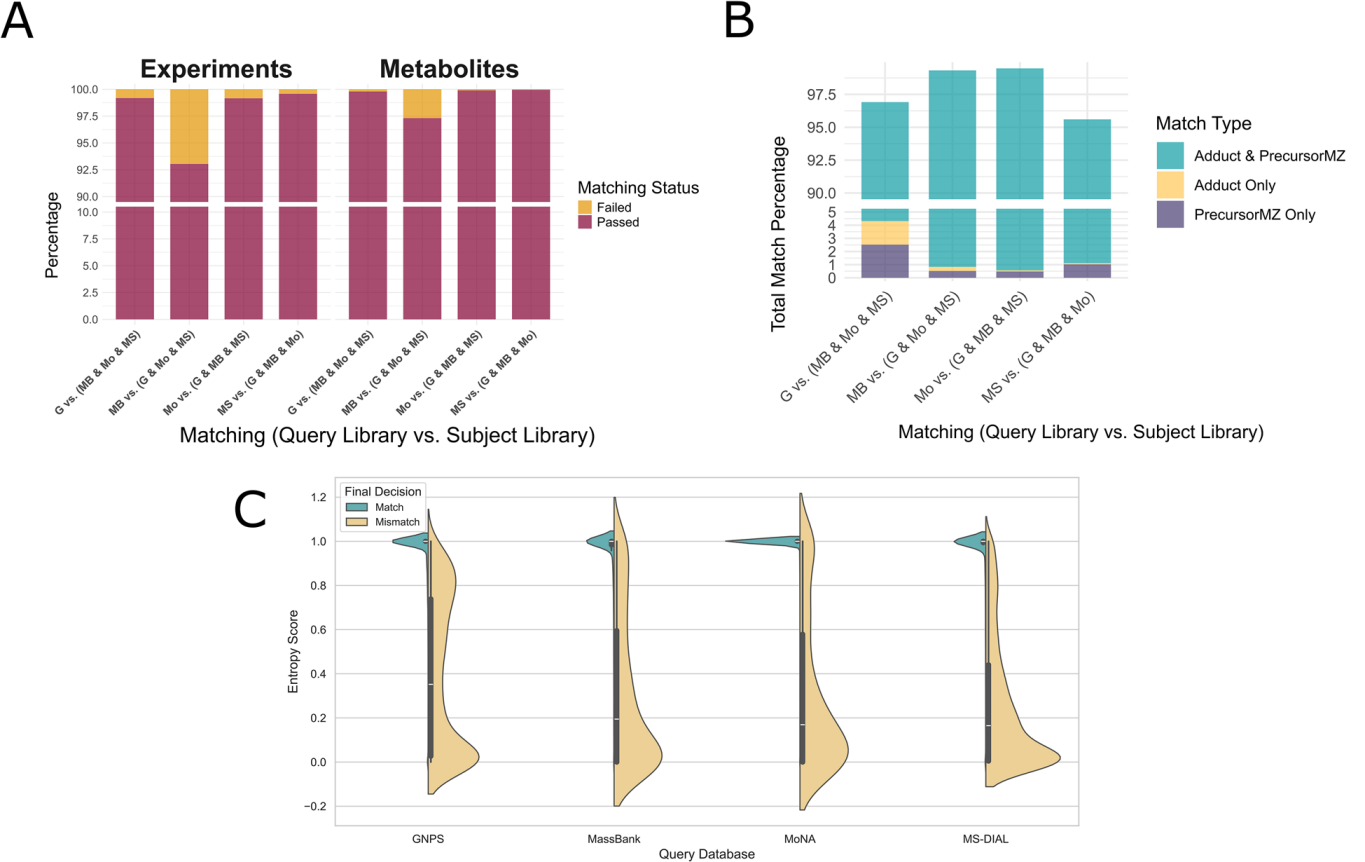
Detailed overview of the first phase of the experiment, which involved comparing each library individually against the integration of the remaining three libraries. **A** Distribution of success and failure outcomes for each spectrum and metabolite. Failures are defined as instances with inconsistent ionization modes or missing subject spectra, often indicating the presence of adducts annotated as UNKNOWN. **B** Stacked bar plot showing the distribution of match types for each library compared against all others. Each bar represents a library, with segments indicating the percentage of matches based on both adduct and precursor m/z, adduct only, or precursor m/z only. **C** Distribution of entropy-based similarity scores in pairwise comparisons across four spectral libraries, stratified by match versus mismatch status. Spectrum pairs were labeled as Match if they satisfied at least one matching criterion—adduct, precursor m/z, or both. Pairs that did not meet any criterion were labeled as Mismatch. The violin and box plots illustrate the distribution of similarity scores across these two groups

As for the first experimental phase, Entropy Similarity results were selected for detailed presentation owing to their demonstrated superior accuracy in the second level of comparison (Fig. 6). Instances of failed matches; defined as spectra lacking a comparable ion mode for spectral matching or an identifiable adduct for match rate determination, constituted less than 0.9% across all comparisons, except for the MassBank comparison, where mismatches represented 6.95% of spectra and 2.66% of distinct metabolites (Fig. 6.A). For the successfully matched spectra, the congruence of both adduct and precursor m/z constituted the most prevalent basis for matches, indicating a low incidence of inconsistencies between these two metadata fields across the libraries. These discrepancies were most pronounced in the GNPS comparison, where 4.15% of spectra were matched based on either the adduct or precursor m/z alone. Such cases likely reflect missing or incorrect precursor m/z values or adduct annotations in either the query or subject spectra (Fig. 6.B). Based on the defined match types, a spectrum pair was considered a match if it shared either the same adduct, the same precursor m/z, or both. Otherwise, it was labeled a mismatch. As expected, most matches showed high similarity scores, reflecting the consistency of spectra from compounds with identical InChIKeys. In contrast, mismatches displayed a wide range of similarity scores, including many high values. This highlights the difficulty of identifying incorrect matches based solely on similarity scores and emphasizes the need for additional metadata filters (Fig. 6.C).

**Fig. 7:**
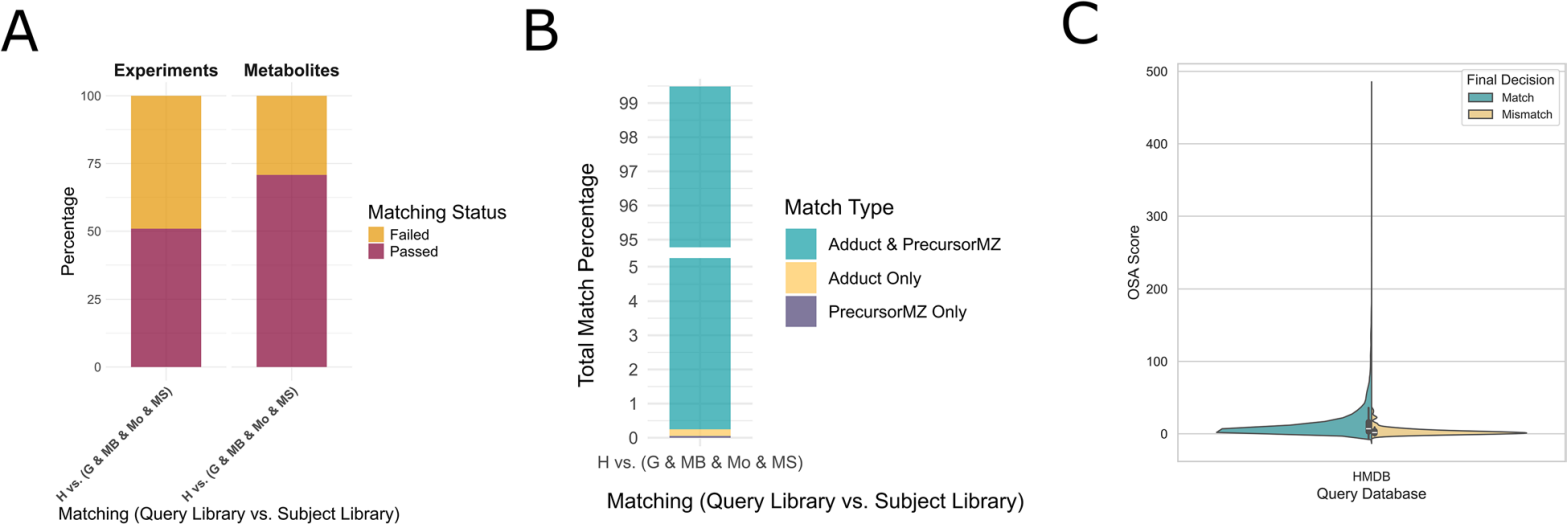
Detailed overview of the second phase of the experiment, in which the HMDB library was compared against the integrated set of the remaining four libraries. **A** Distribution of success and failure outcomes for each spectrum and metabolite. **B** Distribution of match types for each spectrum across all comparisons. **C** Distribution of OSA-based similarity scores in pairwise comparisons in the HMDB, stratified by InChIKey match and mismatch. The violin and box plots show the similarity score distributions for spectrum pairs labeled as Match (identical InChIKey) and Mismatch (different InChIKey)

The second experimental phase involved a pairwise search of the HMDB library against the FragHub spectral library (Fig. 7). The HMDB library comprised 64,140 spectra representing 3975 distinct metabolites. This library was achieved through a curation process that selectively included metabolites with documented metadata on the HMDB website and excluded spectra lacking ion mode annotations. The results of this comparison revealed a significant failure rate affecting a substantial proportion of the HMDB library, reaching 49.06% of the spectra and encompassing 29.18% of the represented metabolites (Fig. 7.A). The primary factor contributing to this failure was the absence of adduct metadata within the HMDB spectra. The observed discordance between the annotated adduct and the experimentally determined precursor m/z was less than 0.25 (Fig. 7.B). The OSA method primarily aims to assign an accurate similarity score for each pairwise comparison between spectra. For each query spectrum, the best match is selected based on the highest similarity score, as reflected in its high match rate. However, it remains challenging to define a fixed score threshold that reliably distinguishes matches from mismatches. This is because mismatched pairs can exhibit similarity scores that closely resemble those of true matches, making it difficult to separate them based on score alone (Fig. 7.C).

**Fig. 8:**
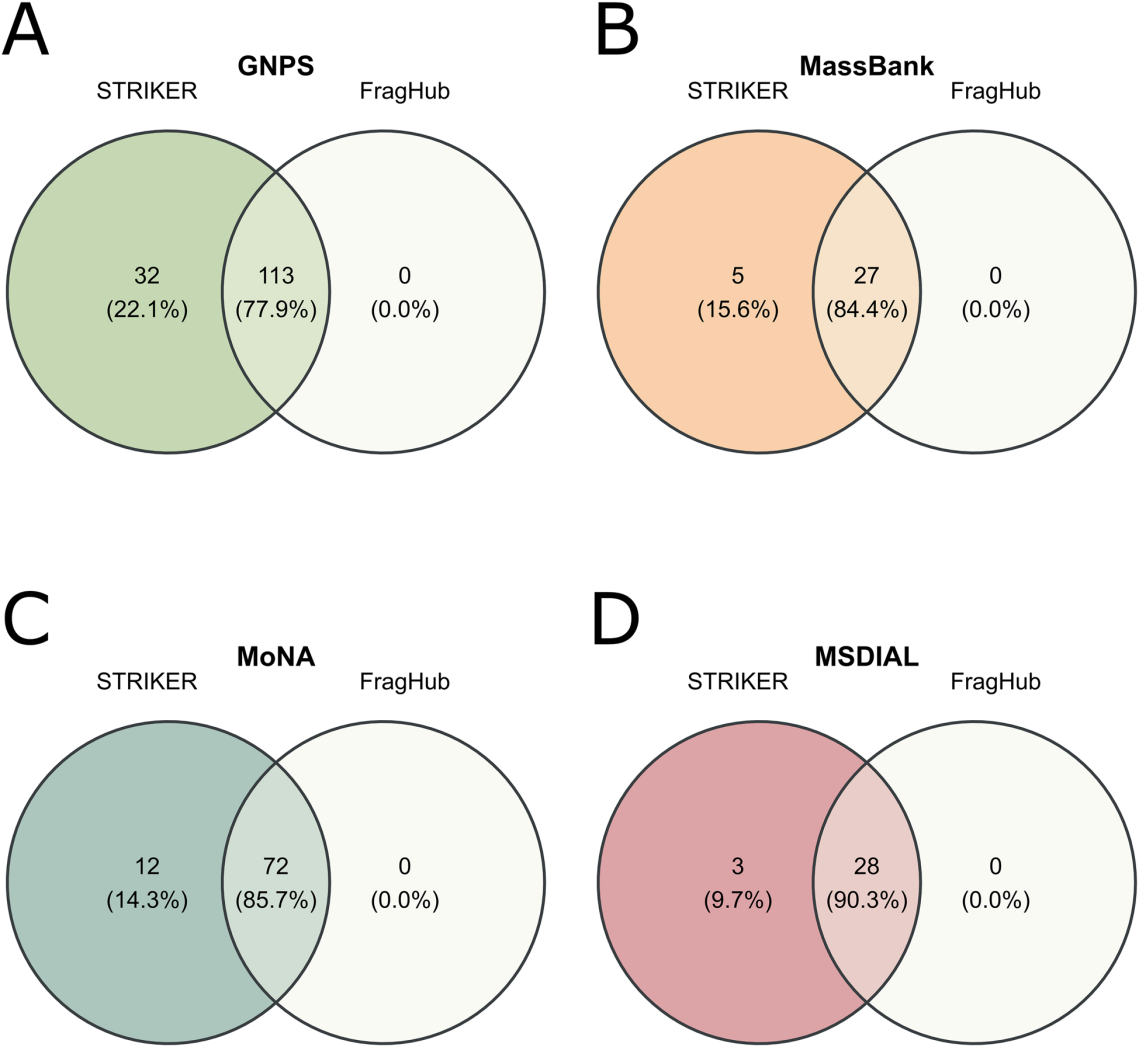
Venn diagrams illustrate the number and percentage of adducts identified by STRIKER and FragHub, highlighting both shared and uniquely detected adducts for each method

To mitigate the heterogeneity of adduct form annotations across spectral libraries, we developed a machine learning pipeline using the MLPClassifier deep learning model, achieving a classification accuracy of 98.91% in mapping diverse adduct representations to a standardized nomenclature (). To quantitatively evaluate the efficacy of this pipeline, we conducted a comparative assessment of STRIKER and FragHub in terms of their capacity to identify adduct forms within benchmark spectral libraries. Our findings demonstrated that STRIKER's enhanced the ability to resolve adduct annotations over FragHub (Fig. 8). For instance, within the GNPS library, STRIKER successfully identified 32 unique adduct forms over the 113 forms commonly identified by both tools (Fig. 8.A). Similarly, in MoNA, STRIKER identified an additional 12 distinct adduct forms (Fig. 8.C). These gains were less pronounced in MassBank and MS-DIAL, which exhibited fewer inconsistencies and a more uniform representation of adduct annotations (Fig. 8.B&D).

## Discussion

Metabolomics has seen substantial growth in both significance and scale, emerging as a powerful approach in single-omics studies as well as in integrative multi-omics research [23]. Untargeted metabolomics, in particular, relies heavily on comprehensive libraries of high-quality spectra. However, the presence of incomplete or erroneous metadata remains a persistent challenge. Rather than correcting these metadata issues, most existing tools discard these spectra, leading to the probable loss of valuable spectral information and potential metabolites. The HMDB is among the libraries most affected by metadata deficiencies. Current metadata cleaning tools exclude nearly half of its spectra (which corresponds to approximately one-third of the library’s metabolites) primarily due to missing adduct and precursor m/z information. To address this issue, we developed STRIKER, a tool designed to correct metadata errors specifically related to adduct annotation. STRIKER predicts the adduct of a given spectrum by comparing it with other spectra acquired under the same ionization mode. This ion mode-based approach is critical for preventing the assignment of incompatible adducts and is supported by the fact that ion mode information is consistently available across spectra in most widely used metabolomics libraries. Consequently, future developments may enable the use of tools that can accurately predict adducts even across differing ionization modes [24]. In addition to the ion mode, matching the spectra to the same compound contributes to increasing the prediction accuracy rate, and therefore we concluded that prioritizing robust compound identifiers is critical. Our analysis showed that while InChIKey and InChI provide consistent representations of chemical identity, SMILES strings can exhibit mismatches if not canonicalized, and molecular formulas or compound names are prone to higher inconsistency due to shared formulas among isomers and ambiguous naming conventions. These findings highlight the importance of using InChIKey or InChI for compound alignment in adduct prediction workflows. A similarity score above 95% was chosen to identify highly similar compounds. However, we observed cases where spectra were 100% similar despite differing compound names or structures, likely reflecting metadata errors for one of the compounds. These findings support the use of ion mode and InChIKey, which improve the accuracy of adduct prediction during compound matching. We employed a two-level experimental framework to benchmark a set of distance-based similarity methods against the most widely used spectral libraries. In the first level of the comparison, it is evident that general similarity metrics are not suitable for adduct prediction, and that similarity metrics specifically developed for spectral comparison must be employed. Furthermore, OSA outperformed other similarity metrics in three out of five comparisons, particularly in the absence of intensity values, demonstrating the effectiveness of OSA in predicting adducts based solely on m/z value similarity. In the second level of the comparison, Entropy Similarity consistently outperformed others, followed by CosineGreedy and OSA. Moreover, Entropy Similarity exhibited the fastest performance, followed by CosineGreedy and OSA; however, OSA demonstrated the highest accuracy in matching adducts within the HMDB library. For OSA, we implemented a dynamic programming approach, as it systematically explores all possible peak alignments within specified tolerance thresholds. Dynamic programming has previously been applied to align peak lists in gas chromatography–mass spectrometry (GC–MS) spectra [25]. In cases of spectral dissimilarity or multiple plausible alignments, we leveraged the distribution of peak lists to identify the optimal match. The city-block distance was selected for its superior performance among the distance metrics. Standardizing adduct formatting remains a significant challenge, primarily because existing tools rely on pattern-matching approaches that are limited in their ability to correct incomplete or improperly formatted adduct annotations. STRIKER addresses this limitation through a pipeline that incorporates an MLPClassifier, achieving correction efficiencies of up to 98%.

**Fig. 9:**
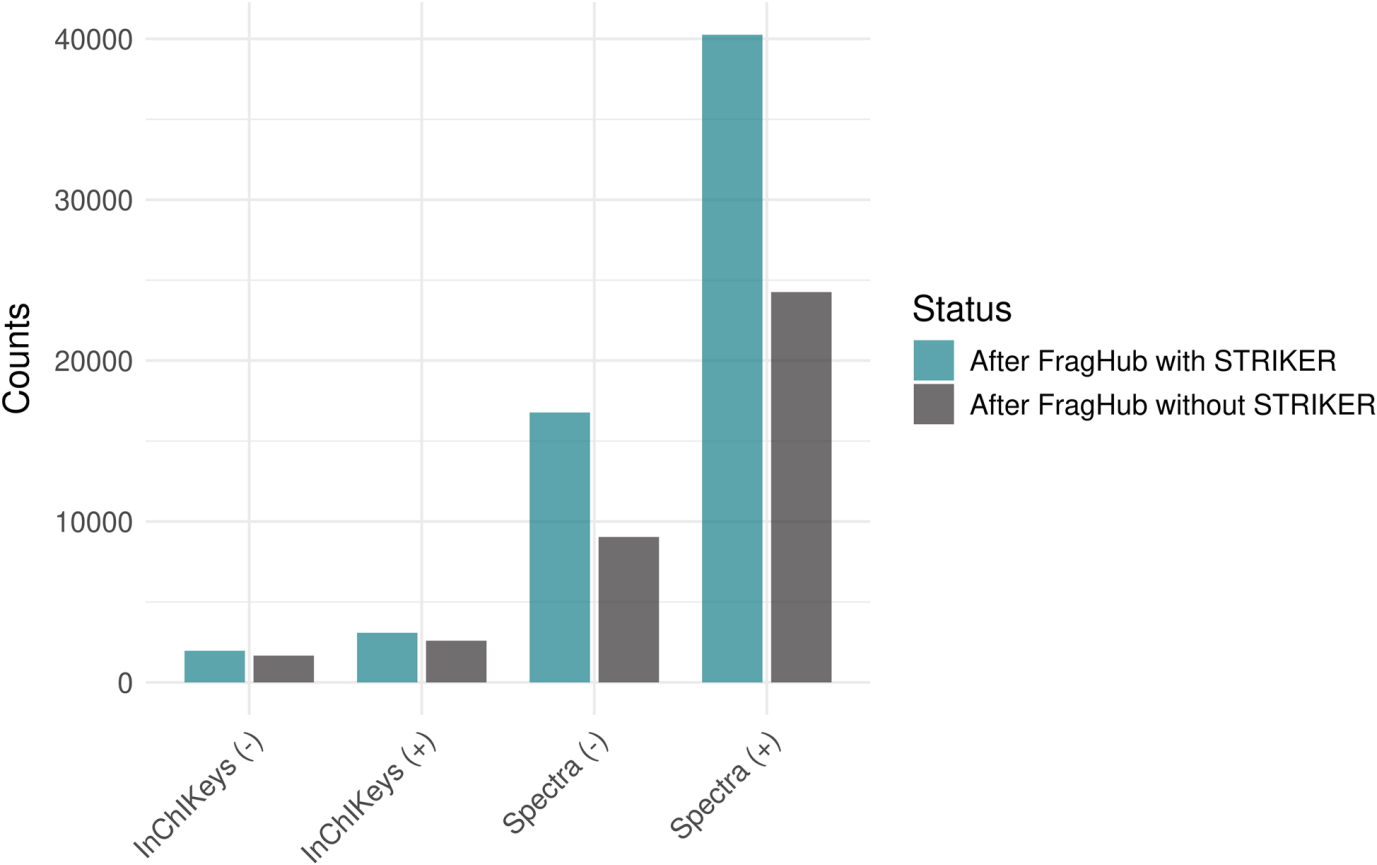
Number of spectra and unique InChIKeys obtained with and without the application of STRIKER

We utilized FragHub to refine the HMDB library under two conditions: without STRIKER and with STRIKER. In the first approach, FragHub was applied directly to the unmodified HMDB library, resulting in 24,257 spectra in positive ion mode and 9035 spectra in negative ion mode, corresponding to 2590 unique InChIKeys in positive mode and 1663 in negative mode. In contrast, the second approach involved first using STRIKER to correct adduct annotations in the GNPS, MassBank, MoNA, and MS-DIAL libraries. These corrected libraries were then integrated to form a comprehensive reference set. STRIKER was subsequently used to predict and standardize adducts in HMDB by comparison with the integrated library. This STRIKER-enhanced processing yielded 40,254 spectra in positive mode and 16,774 spectra in negative mode, corresponding to 3082 InChIKeys in positive mode and 1965 in negative mode (Fig. 9).

As a result, when working with publicly available spectral libraries, STRIKER is recommended for comprehensive adduct correction—both in terms of normalizing adduct formatting and predicting missing adducts. This dual approach substantially reduces the number of spectra that would otherwise be excluded by metadata normalization or cleaning tools. STRIKER generates the HMDB spectral library in MSP format by downloading and integrating multiple data files available from the HMDB website. It also supports the extraction of sublibraries from larger datasets, enabling the creation of specialized, application-specific libraries. Designed with accessibility in mind, STRIKER features a user-friendly Python-based GUI, making it suitable for researchers without a bioinformatics background.

## Conclusions

Overall, STRIKER aims to enhance the comprehensiveness and utility of metabolite spectral libraries annotation in untargeted metabolomics studies by correcting and matching adducts in spectra with errors or missing annotations. Its intuitive interface and streamlined design make it particularly accessible to researchers without bioinformatics expertise, allowing them to work with spectral libraries directly without being burdened by formatting handling. STRIKER is intended to serve as a foundational preprocessing step—before metadata cleaning and normalization—improving both the quality and completeness of spectral libraries.

## Limitations of the work

Although STRIKER effectively predicts missing adducts using spectral similarity, ion mode, and InChIKey matching, there are still a few limitations. While users can define custom similarity score thresholds for entropy and CosineGreedy methods, the interpretation of these scores can vary depending on the input data and subject library composition. Additionally, the Optimized Spectral Alignment (OSA) method does not rely on a traditional similarity score threshold. No default threshold is recommended for OSA, as it is designed specifically for use with HMDB and applies strict filtering based on ion mode and InChIKey agreement. Another key limitation of this work lies in the missing or non-standardized metadata in public spectral libraries, particularly instrument type and collision energy. These factors significantly influence fragmentation patterns and, consequently, method performance. Since such metadata is often unreliable, STRIKER relies on accuracy testing to guide method selection rather than metadata-based assumptions. Another limitation relates to the spectral library, where discrepancies may occur between the assigned structure and the spectrum. In such cases, the spectrum may be matched to an incorrect compound, which may in turn lead to inaccurate adduct prediction.

## Supplementary Information

Supplementary Material 1.

Supplementary Material 2.

Supplementary Material 3.

## Data Availability

Project name: STRIKER. Project home page: https://striker-gui.sourceforge.io. Operating system(s): Windows, Linux, macOS. Programming language: Python. User interface: GUI and Python package. Documentation: GUI documentation provided as a PDF within the downloadable package and accessible through the “About STRIKER” tab in the GUI; Python package documentation available at https://striker-gui.sourceforge.io/docs/. License: MIT License Declarations. Availability of data and materials: The source code of STRIKER is available at https://sourceforge.net/p/striker-gui/code/. STRIKER is also available as a Python package at https://striker-gui.sourceforge.io, https://striker-gui.sourceforge.io/docs/.
