# Supplementary material for: STRIKER: a spectral metadata repairing tool for expanding the comprehensiveness of spectral libraries
Source: J Cheminform. 2026 Jan 27;18:26. doi: 10.1186/s13321-026-01150-4 (PMC12918191; doi:10.1186/s13321-026-01150-4)
Supplement: Supplementary file 1 — Supplementary Material 1. [file 13321_2026_1150_MOESM1_ESM.docx]

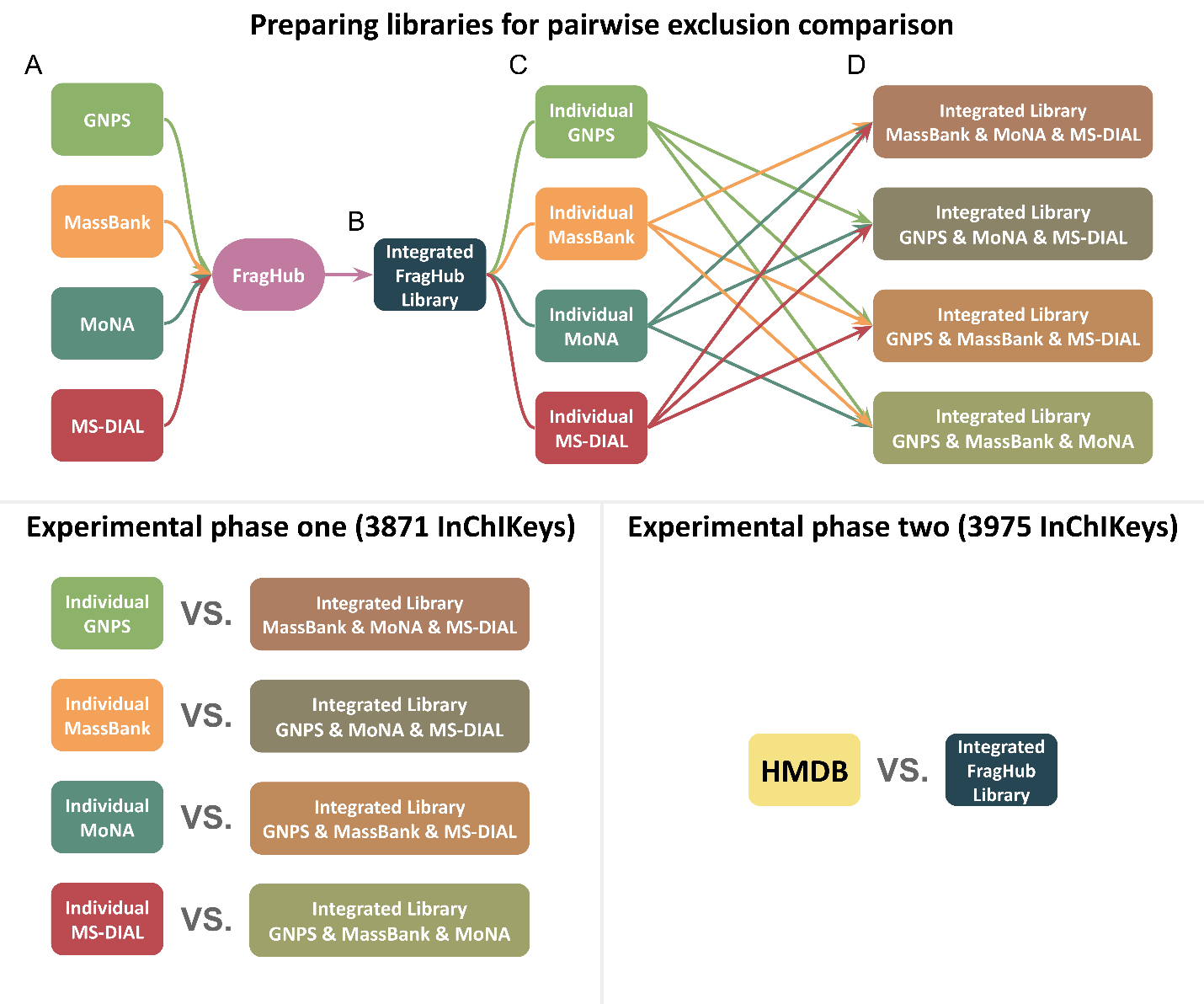


**Figure S1. Description of data preparation for benchmark and phase comparison experiments. (A) The four spectral libraries used in this study. (B) The integrated FragHub library generated by applying FragHub to merge and normalize metadata across the four libraries. (C) The four normalized spectral libraries individually extracted from the integrated library to serve as query libraries for pairwise comparisons. (D) The subject libraries used in comparisons, each constructed by integrating three of the four normalized libraries to enable one-against-rest evaluations. In experimental phase one, spectra shared among the four libraries were identified based on InChIKeys to avoid inconclusive and computationally intensive comparisons. Each library was then individually compared against an integrated library composed of the other three. In experimental phase two, the HMDB library was compared directly with the integrated FragHub library.**


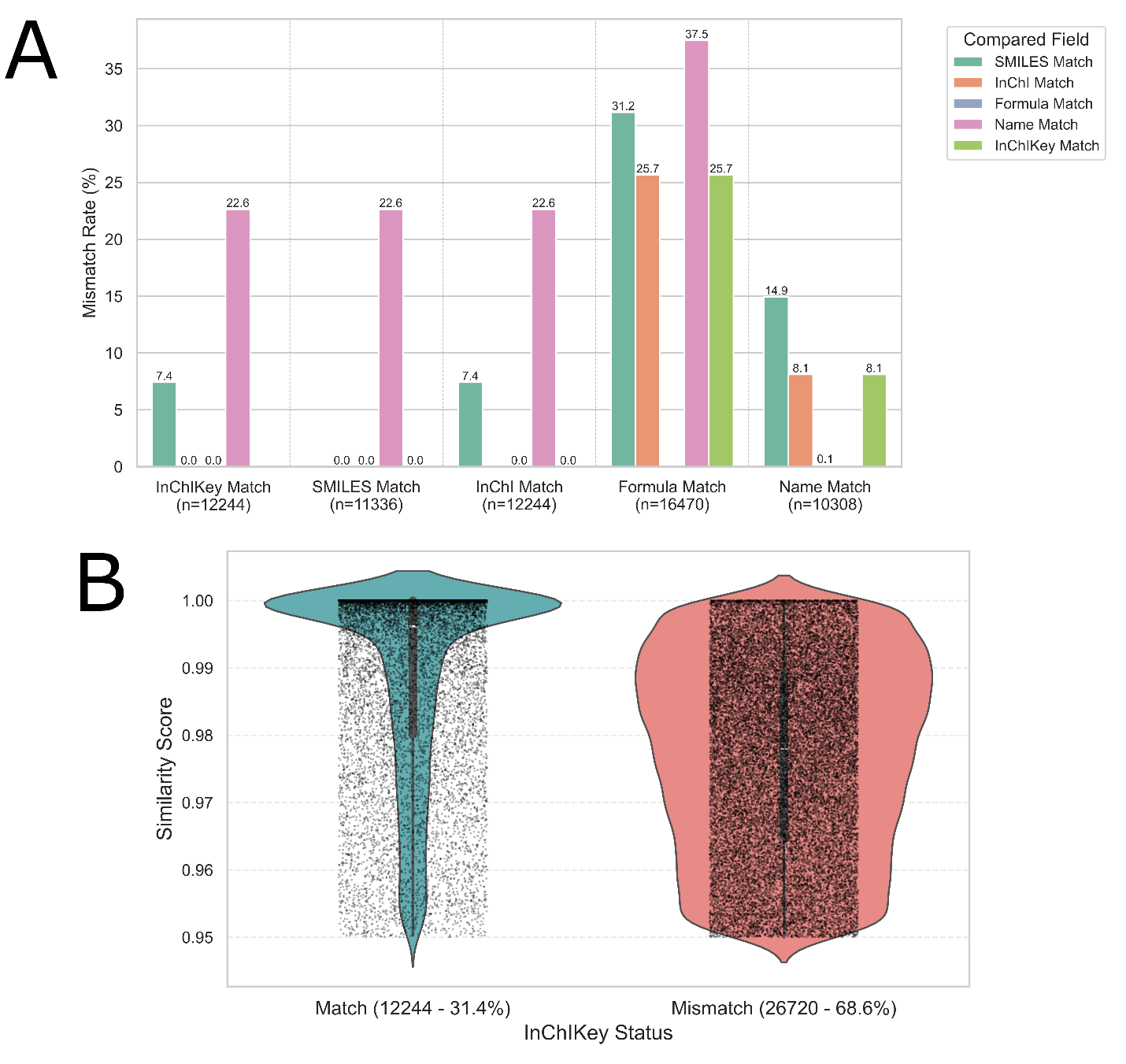


**Figure S2. Metadata assessment in OMSLs. (A) Error rate across metadata fields when a specific metadata field is identical between query and subject spectra. For example, in the first element on the X-axis (InChIKey), we calculated the percentage of mismatches in all other metadata fields among spectrum pairs with identical InChIKeys. (B) Similarity score for the best score hit for each query spectrum, divided into Match (query and subject share identical InChIKeys) and Mismatch (query and subject do not share identical InChIKeys). High mismatch rates are observed even at similarity scores between 0.95 and 1.0, highlighting the limitation of relying on fixed thresholds to define correct spectral matches.**


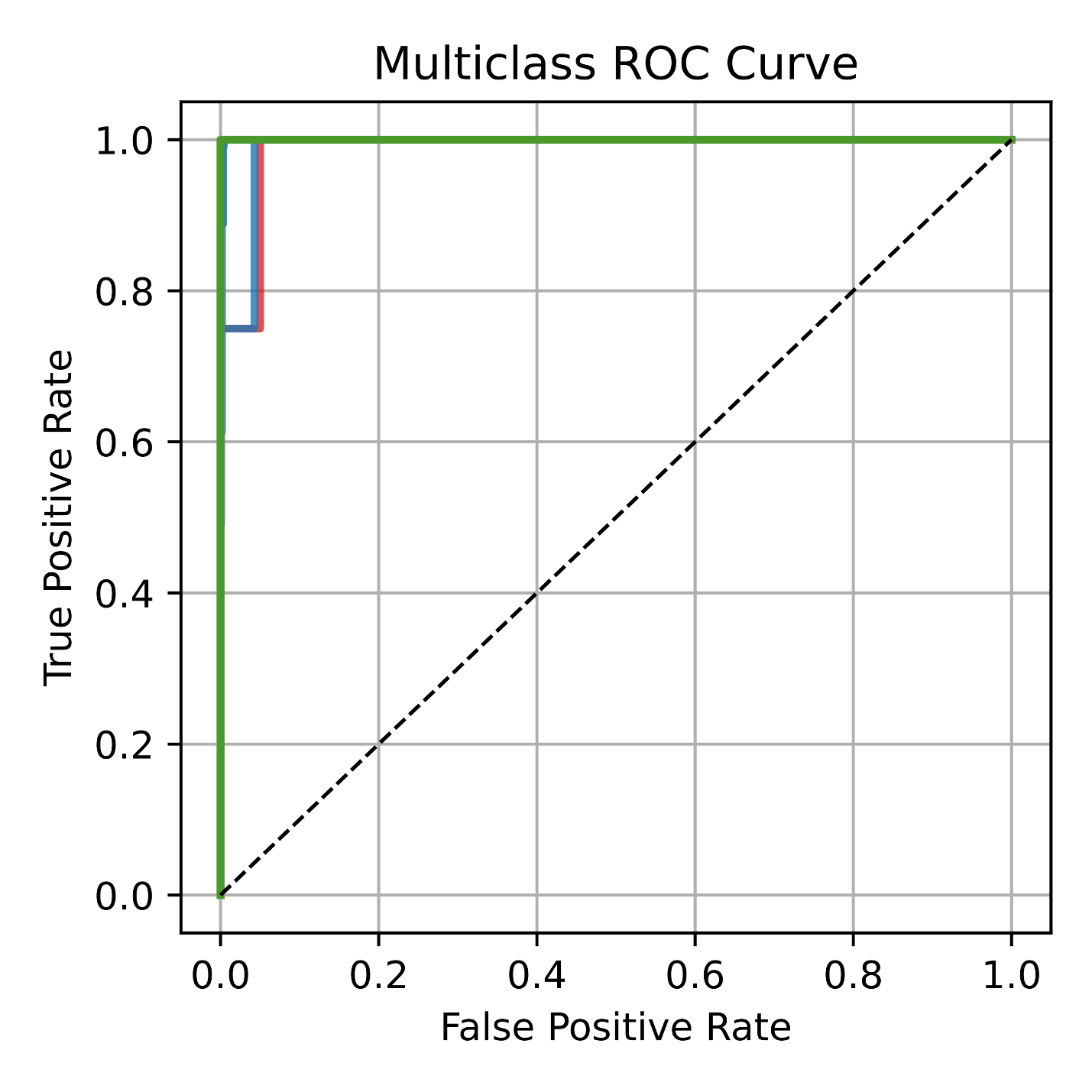


**Figure S3. ROC curves illustrating the accuracy of adduct classification across multiple adduct types.Each curve reflects the model's ability to correctly identify and correct specific adduct forms.**
